# Supplementary material for: Synthesis, Characterization and Non-Isothermal Crystallization Kinetics of a New Family of Poly (Ether-Block-Amide)s Based on Nylon 10T/10I
Source: Polymers (Basel). 2020 Dec 27;13(1):72. doi: 10.3390/polym13010072 (PMC7795608; doi:10.3390/polym13010072)
Supplement: Supplementary file 1 [file polymers-13-00072-s001.pdf]

# Supporting Information

## Synthesis, Characterization and Non-isothermal Crystallization Kinetics of a New Family of Poly (ether-block-amide)s Based on Nylon 10T/10I

Xin Tong [a], Zhao Wang [a], Mei-Ling Zhang [b], Xiao-Jun Wang[*b,c], Gang Zhang [b], Sheng-Ru Long [b], Jie Yang[*b,d]

[a]College of Polymer Science and Engineering, Sichuan University, Chengdu 610064, People's Republic of China

[b]Analytical and Testing Center, Sichuan University, Chengdu 610064, People's Republic of China

[c]State Key Laboratory of Organic–Inorganic Composites, Beijing 1000294, People's Republic of China

[d]State Key Laboratory of Polymer Materials Engineering, Sichuan University, Chengdu 610065, People's Republic of China

Correspondence to: XJ Wang (wangxj@scu.edu.cn) or J. Yang (E-mail: ppsf@scu.edu.cn)

**Figure S1**. The stress versus strain of TPAE-1000 and Pebax® 5533 at room temperature 25 °C............................................................................................................................................S3
**Figure S2.** High temperature bending test: (a) TPAE-1000; (b) TPAE-1500; (c) TPAE-2000; (d) Pebax®5533..........................................................................................................................S3
**Figure S3.** POM micrographs of (a)TPAE-1000, (b) TPAE-1500, (c) TPAE-2000 and (d) PA10T/10I nonisothermally crystallized at 5 °C/min..................................................................S4
**Figure S4.** POM micrographs of (a)TPAE-1000, (b) TPAE-1500, (c) TPAE-2000 and (d) PA10T/10I nonisothermally crystallized at 20 °C/min................................................................S4

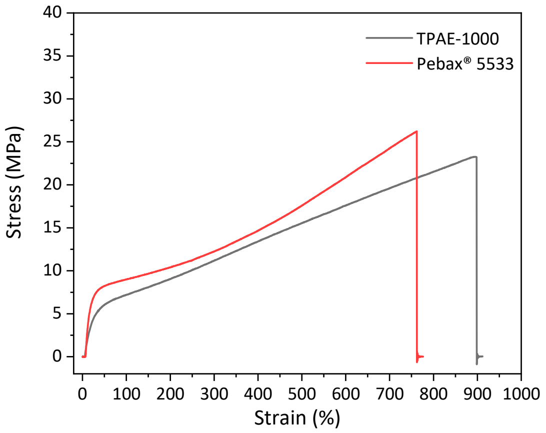


**Figure S1**. The stress versus strain of TPAE-1000 and Pebax® 5533 at room temperature 25 °C

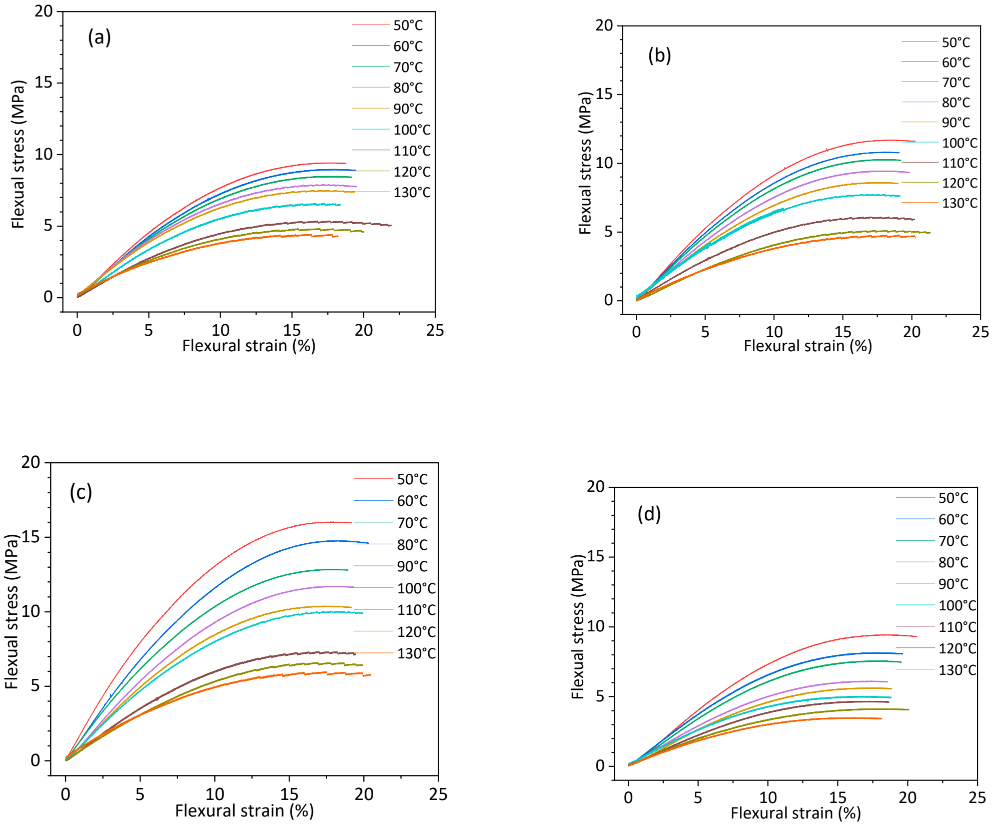


**Figure S2.** High temperature bending test: (a) TPAE-1000; (b) TPAE-1500; (c) TPAE-2000; (d) Pebax® 5533

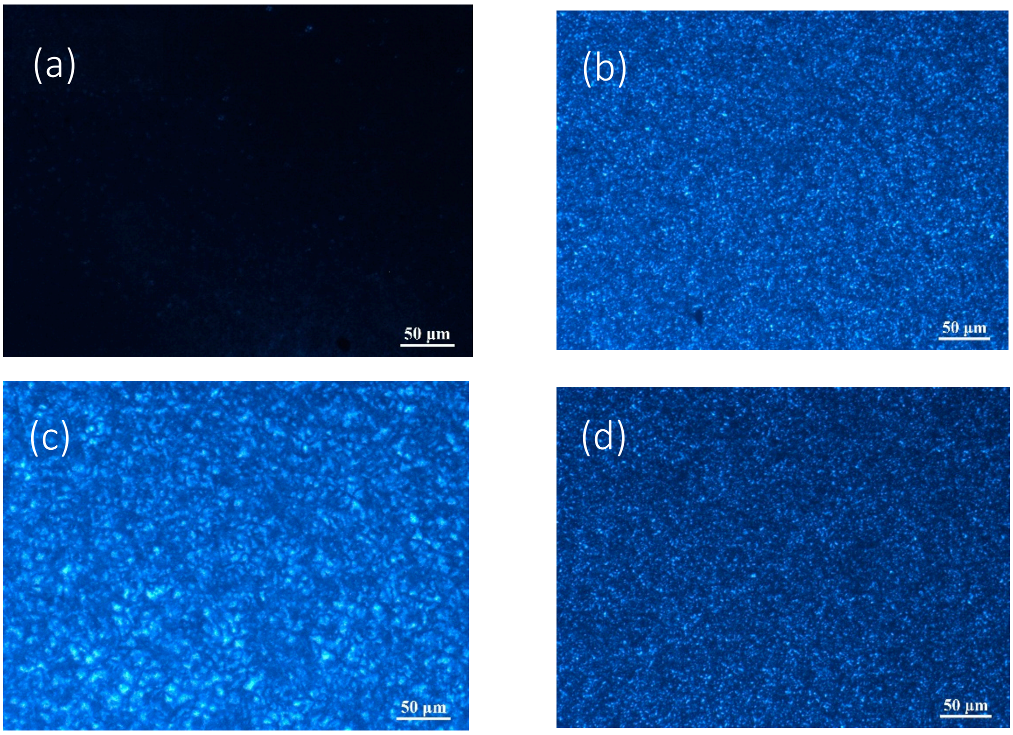


**Figure S3.** POM micrographs of (a)TPAE-1000, (b) TPAE-1500, (c) TPAE-2000 and (d) PA10T/10I nonisothermally crystallized at 5 °C/min

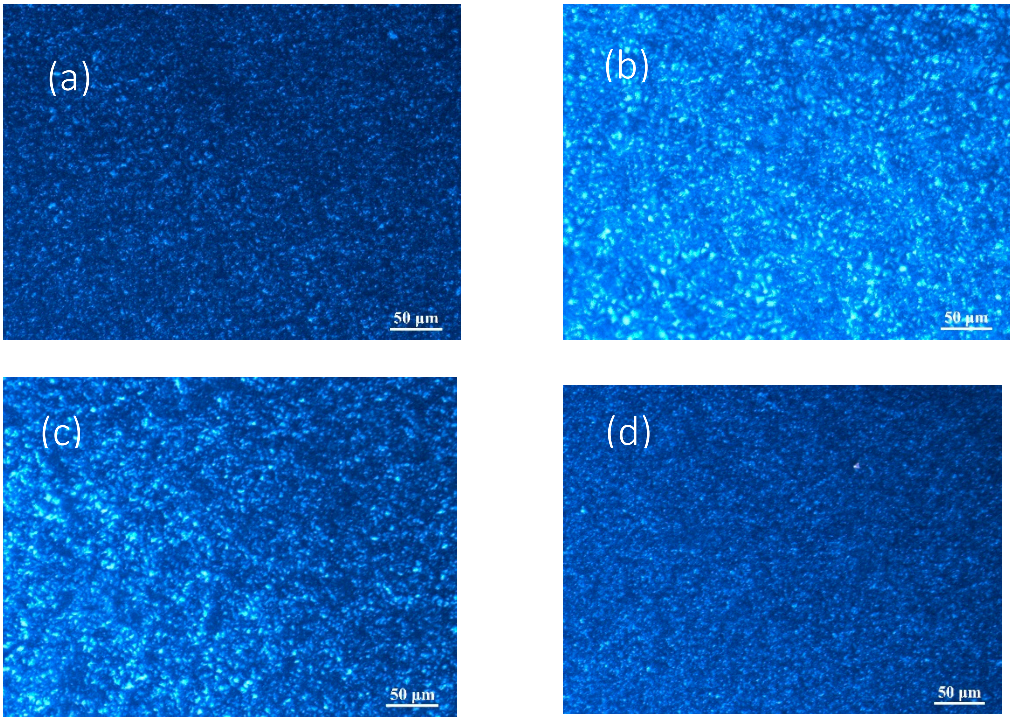


**Figure S4.** POM micrographs of (a)TPAE-1000, (b) TPAE-1500, (c) TPAE-2000 and (d) PA10T/10I nonisothermally crystallized at 20 °C/min
